# Melatonin mitigates chemotherapy-induced small intestinal atrophy in rats and reduces cytotoxicity in murine intestinal organoids

**DOI:** 10.1371/journal.pone.0307414

**Published:** 2024-09-03

**Authors:** Karsten Peters, Ada Lerma Clavero, Fredrik Kullenberg, Maria Kopsida, David Dahlgren, Femke Heindryckx, Hans Lennernäs, Markus Sjöblom

**Affiliations:** 1 Department of Medical Cell Biology, Uppsala University, Uppsala, Sweden; 2 Department of Pharmaceutical Biosciences, Uppsala University, Uppsala, Sweden; University College London Institute of Child Health, UNITED KINGDOM OF GREAT BRITAIN AND NORTHERN IRELAND

## Abstract

Cancer continues to pose a significant global health challenge, with gastrointestinal (GI) cancers among the most prevalent and deadly forms. These cancers often lead to high mortality rates and demand the use of potent cytotoxic chemotherapeutics. For example, 5-fluorouracil (5-FU) forms the backbone of chemotherapy regimens for various GI cancers, including colorectal cancer. While these chemotherapeutics efficiently kill cancer cells, they frequently cause off-target effects such as chemotherapy-induced mucositis (CIM), characterized by debilitating symptoms like pain, nausea, and diarrhoea, necessitating medical intervention. In this study, we elucidated the potential of melatonin and misoprostol to reduce 5-FU-induced small intestinal mucositis. Morphological and cellular changes in the jejunum, along with colonic faecal water content were quantified in rats as markers for CIM. Additionally, the effects of melatonin were investigated *in vitro* on 5-FU treated murine intestinal organoids. The results showed that melatonin prevented villus atrophy in the rat jejunal mucosa and upheld cell viability in murine intestinal organoids. In contrast, misoprostol alone or in combination with melatonin did not significantly affect CIM caused by 5-FU. These *in vivo* and *in vitro* experiments provided promising insights that melatonin may be used as a preventive and/or adjuvant combination therapy to prevent and reduce CIM, holding the potential to enhance cancer treatment outcomes and improve patient quality-of-life.

## Introduction

Cancer remains a leading global cause of premature death [1], with its impact projected to increase due to an expected decrease in mortality from stroke and cardiovascular disease [2]. Gastrointestinal (GI) cancers, among the most prevalent and deadly forms, significantly contribute to this global health burden. While significant progress has been made in cancer treatment through the introduction of targeted therapies such as monoclonal antibodies, antibody-drug conjugates, or immunotherapy [3], cytotoxic chemotherapeutics are still widely used due to their versatility and cost-efficiency [4]. Chemotherapeutics exert their anti-cancer effect by targeting intracellular mechanisms and signalling pathways in rapidly proliferating cells. In parallel, these cytotoxic effects are general and often lead to toxicity in rapidly proliferating normal tissues, such as the myeloid and lymphoid tissues, gonads, and the intestinal epithelium [5]. The fluoropyrimidine 5-fluorouracil (5-FU) is an antimetabolite agent that forms the backbone of chemotherapy regimens for various cancers, including gastrointestinal cancers, as well as head and neck cancers [6]. 5-FU exerts its cytotoxic effects through inhibition of thymidylate synthase (TS), generation of reactive oxygen species (ROS), and incorporation of its metabolites into RNA and DNA in both malignant and normal proliferating cells. In the GI tract, the off-target toxicity of chemotherapy induces mucositis, a complex toxicity that affects almost all patients treated with chemotherapeutics, depending on the agent(s) and dose used [7]. The symptoms of chemotherapy-induced mucositis (CIM) include pain, ulceration, and difficulty swallowing, leading to a severe reduction in the quality of life in patients. The breakdown of the mucosal barrier due to CIM can result in serious complications such as fever, sepsis, and multi-organ dysfunction, which may potentially be fatal. CIM is known to cause grade 3 to 4 diarrhoea in one third of patients, which is defined as more than seven bowel movements above normal per day and subsequent hemodynamic disturbances [8]. Preventing these symptoms often requires dose-reduction or discontinuation of cancer treatment, which in turn reduces efficacy of the anti-tumour response and may affect the therapeutic outcome. Subsequently, management of these symptoms is associated with substantial health care costs from extra consultations, emergency room visits and hospitalizations [9, 10]. It is therefore necessary to explore novel effective supportive and preventive treatments for CIM.

The importance of enteroendocrine feedback signalling when quantifying GI toxicity and recovery following chemotherapy disqualify the translational relevance of many simple *in vitro* two-dimensional (2-D) intestinal models. More suited are *in vivo* models or more complex *in vitro* systems [11, 12]. One established *in vivo* CIM model is the rat [13] which has been used in several of our earlier studies evaluating diarrhoea [14], permeability [15, 16], and supportive treatments [17].

Intestinal organoids, also known as enteroids or “mini-guts”, are three-dimensional (3-D) structures derived from stem cells that closely recapitulate the architecture and function of the small intestine [18, 19]. These organoids are typically generated from mouse small intestinal tissue and serve as robust tools for mechanistic investigations of intestinal development, physiology, and pathophysiology [20]. They form 3-D structures that consist of a central lumen surrounded by a polarized epithelial layer containing various differentiated cell types, including absorptive enterocytes, goblet cells, enteroendocrine cells, and paneth cells [18, 20]. One prominent morphological characteristic of mouse intestinal organoids is their display of multiple crypt-like structures that extend from the central lumen that replicate the crypt-villus units observed in the intestinal epithelium [20, 21]. Studies have shown that organoids mimic the cellular diversity of the intestinal epithelium, including key cell types such as enterocytes, goblet cells, Paneth cells, and enteroendocrine cells [22, 23]. These cells play vital roles in nutrient absorption, mucus production, antimicrobial peptide secretion, and hormone release [24], making organoids highly relevant for modeling intestinal physiology and disease. They outperform traditional cell lines in sensitivity to inflammatory stimuli, providing responses that closely resemble the *in vivo* environment [25]. This makes organoids a robust model system for studying intestinal barrier function and the effects of various stimuli on epithelial integrity, enabling meaningful investigations into CIM and potential interventions [22, 23].

Melatonin is a hormone released from the pineal gland that is associated with a multiplicity of physiological actions, such as the control of the circadian rhythm for sleep and core temperature [26], immunoregulation [27], and protection from oxidative and inflammatory damage [28]. The pineal gland is the primary source of systemic melatonin, which circulates throughout the body and mediates these wide-ranging effects. In contrast, the GI mucosa is also a significant source [29], where it is synthesized by enterochromaffin cells [29]. Unlike the photoperiodically regulated production of melatonin in the pineal, the release of melatonin from the intestine seems to be related to the periodicity of food intake [30] and may significantly contribute to circulating concentrations of melatonin, especially during daytime [29].

Local effects of melatonin in the GI tract include the reduction of ethanol- and radiation-induced increases in mucosal permeability [31, 32], increasing duodenal mucosal bicarbonate secretion and reducing basal jejunal mucosal permeability. While many of its effects are receptor mediated [33, 34], melatonin is also known to be a potent scavenger of ROS [35]. Melatonin directly scavenges a variety of ROS, including hydroxyl radicals (^•^OH), hydrogen peroxide (H_2_O_2_), and singlet oxygen (^1^O_2_). The indole ring structure of melatonin allows it to donate electrons to these reactive molecules, converting them into less harmful forms and thus preventing oxidative damage to cellular components such as lipids, proteins, and DNA [36]. In addition, the antioxidant protection of melatonin is correlated both to its own redox active properties and to metabolites originated during its metabolism. Melatonin stimulates the activity of several key antioxidative enzymes, such as glutathione peroxidase and glutathione reductase [35, 37]. These enzymes play a critical role in the detoxification of hydrogen peroxide and the maintenance of the cellular antioxidant glutathione in its reduced form. These combined actions make melatonin and its metabolites a critical agent in mitigating oxidative stress and protecting cellular integrity, which is central to its therapeutic potential in conditions characterized by excessive ROS production, including chemotherapy-associated off-target toxicities and CIM [24].

Misoprostol is a small E-type prostaglandin analogue that inhibits production and release of pro-inflammatory cytokines such as interleukin 1 [38, 39] and interleukin 6 [40], which are mediators of inflammatory signalling and cytokine storms involved in the progression of CIM [41]. Initially developed to prevent and treat gastric ulcers, particularly those induced by non-steroidal anti-inflammatory drugs (NSAIDs), misoprostol decreases gastric acid secretion, increases intestinal mucus and bicarbonate secretion, and maintains mucosal blood flow [42]. These actions contribute to the formation of a protective barrier that shields epithelial cells from gastric acid and other irritants, thereby maintaining the integrity of the intestinal mucosal lining and preventing ulcer formation [43, 44]. Combined with its anti-inflammatory properties, misoprostol might be relevant in slowing down the progression of CIM by mitigating the inflammatory response and protecting the gastrointestinal mucosa [45].

The main objective of this study was to investigate the role of melatonin, alone or in combination with misoprostol, on 5-FU-induced *in vivo* jejunal mucosal morphological, cellular changes and faecal water content. The effects of melatonin on 5-FU-induced reduced cell viability were examined in the 3D intestinal organoid mouse model.

## Material and methods

### Chemicals and solutions

Accustain formalin solution (10%, neutral buffered), ethanol, 5-ethyl-5-(1′-methyl-propyl)-2-thiobarbiturate (Inactin), dimethyl sulfoxide, melatonin and phosphate buffered saline tablets (PBS, pH 7.4) were purchased from Sigma-Aldrich (St. Louis, MO, USA). Sodium phosphate dibasic dihydrate (Na_2_HPO_4_∙2H_2_O), potassium dihydrogen phosphate (KH_2_PO_4_), sodium hydroxide, methanol and sodium chloride were purchased from Merck KGaA (Darmstadt, Germany). Invitrogen RNAlater Stabilization Solution was purchased from Fisher Scientific (Pittsburgh, PA, USA). All solvents were HPLC-grade or higher and water was of ultra-pure grade (Milli-Q). 5-Fluorouracil (5-FU) was purchased from Apoteket AB (Sweden). Misoprostol was purchased from Tocris Bioscience (Bristol, UK) Transferrin Ki67 antibody (RRID:AB_302459), horseradish peroxidase–DAB (3,3’-diaminobenzidine) Detection IHC Kit (RRID:AB_2810213) and terminal deoxynucleotidyl transferase dUTP nick-end labelling (TUNEL) Assay Kit–HRP-DAB (RRID:AB_2925017) were purchased from Abcam (Cambridge, UK). Inactin was prepared at 500 mg/ml in deionised water. The stock solution was then diluted to 5 mg/ml in physiological saline, which was the administered concentration.

### Animals

#### Rats

This animal *in vivo* study was approved by the local ethics committee for animal research (Dnr 5.8.18-06777/2020) in Uppsala, Sweden. We used the Uppsala University extended criteria for animal welfare for rodents and rabbits. A total of 0.8 points on this scale was our humane endpoint. No animals in this study reached this endpoint. The animal health and behaviour were monitored twice daily. All staff involved in handling the animals were special trained in animal care or handling at a minimum of FELACA C. Conventional male Wistar Han IGS rats (strain code 273) from Charles River Co. (Germany) with body weights from 280–470 g (8–14 weeks old) were used. All animals [in total 36 rats] were allowed to acclimatise for at least one week in the Animal Department, Uppsala University, prior to the start of the experiment and allowed water and food *ad libitum*. Housing conditions were 21–22°C at a 12–12 hour light-dark cycle with 60% relative humidity. The total number of rats included in the study was 36. As a means to reduce the number of animals used for this study, the data of 12 animals (control group and 5-FU alone) was used both in this and a separate, parallel study run in our lab.

#### Mice

This method was approved by the Uppsala ethical committee for animal experimentation (DNR 5.8.18-0089/2020) and RESIST-guidelines were followed. One conventional male Sv129-mice (129S2/SvPasCr) from Janvier-Labs (France) was used for the organoid isolation. Before isolation of organoids the mice were sacrificed by cervical dislocation.

#### Rat study design

**Fig 1:**
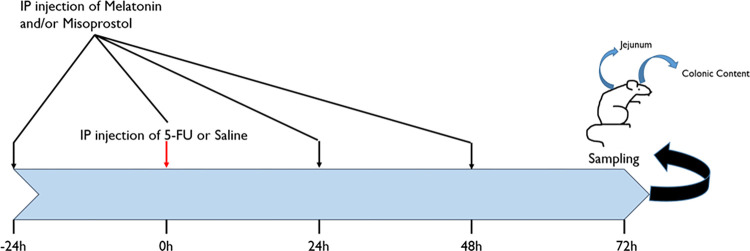
A schematic outline of the experimental design for the six study groups (n = 6 in each group). In the first group, saline was given once at time point 0 h (control). In 4 groups, gastrointestinal (GI) toxicity was induced by a single intraperitoneal (IP) injection of 5-Fluorouracil (5-FU, 200 mg/kg) at time point 0 h. In 3 of these 5-FU groups, a treatment with melatonin (10 mg/kg) and/or misoprostol (1 mg/kg) was given once a day (OD) for 4 days (-24 to 48 h). In the last group, the combination of melatonin and misoprostol was given once daily for 4 days but without any induction of GI toxicity with 5-FU. At 72 h, the rats were sedated and samples were collected.

The overall *in vivo* study design of the rat study is schematically displayed in Fig 1. The study included six groups with six animals in each where all dosing with all drugs was conducted intraperitoneally (IP). Two groups were not dosed 5-FU: one single-dose saline (Control), one exposed to melatonin and misoprostol once daily during four days. One group was dosed with 5-FU only at t = 0, and three groups also together with a daily dose of melatonin and/or misoprostol starting at t = -24. At t = 72 hrs study was terminated and the rats were anesthetised (Inactin IP, 180 mg/kg). The full colonic content was retrieved, weighed and dried, while jejunal tissue samples were taken for morphological, proliferative and apoptotic analysis (see section 2.4). During deep anaesthesia induced with inactin the rats were sacrificed by an intravenous injection of saturated potassium chloride.

The doses and administration route of melatonin (10 mg/kg IP daily), misoprostol (1 mg/kg IP daily) and 5-FU (200 mg/kg IP only as single dose) were selected based on previous experience with melatonin and misoprostol [33, 45–47] and for clinical translation based on standard doses for 5-FU [48]. The saline and 5-FU control groups in this study were the same as in one of our earlier preclinical studies that was run in parallel [14]. This study follows the ARRIVE guidelines and the study protocol established before the study included information on rat ID and weight, dose(s), dosing time and volumes, sampling times, samples, and sample handling. A humane end-point was defined and animal well-being was monitored twice daily to avoid unnecessary suffering among the included animals. A weight loss of >20% was set as a criterion for early termination of the study.

#### Sample collection and handling

After sedation, rats were dissected and tissue samples were taken from the jejunum for histological analyses. These tissue samples were immediately fixed in 10% formalin for 24 h, after which they were moved to 70% ethanol and then embedded in paraffin for subsequent histological and immuno-histological analyses. The colon was removed in its entire length and faecal contents were weighed and then thereafter placed in an oven at 50°C for up to three days, and the dried content was then weighed again. The percent colonic water content, our marker for diarrhoea, was calculated as (wet weight—dry weight) / wet weight × 100.

#### Murine intestinal organoids isolation and culture

Isolation and culture of murine intestinal crypts was performed following manufacturer´s guidelines of IntestiCult™ Organoid Growth Medium (Mouse) from Stemcell (Stemcell, 06005). Geltrex™ LDEV-Free Reduced Growth Factor Basement Membrane Matrix (ThermoFisher, A1413202) was used for maintenance of organoid cultures [23]. IntestiCult™ Organoid Growth Medium (Mouse) was supplemented with 1% penicillin/streptomycin (ThermoFisher, 15140122) and changed every 2–3 days. Organoid cultures were passaged every 7–10 days with an average split ratio of 1:6 and maintained in an incubator at 37^∘^C, 5% CO_2_, and 21% O_2_.

#### Cell viability assay

After 7 days in culture, small intestinal organoids were pre-treated with melatonin (0.1 mM) overnight. The next day a stock solution of 5-FU (1 x 10^5^ μM) was prepared in fresh growth medium and serially diluted 1:10. Subsequently, organoids were treated with increasing concentrations of 5-FU (from 1 x 10^−3^ to 1 x 10^5^ μM) ± melatonin (0.1 mM) for 24 hours. After 24 hours, cell viability was measured using the CellTiter-Glo® 3D Cell Viability ATP-based assay (Promega, G9683) [49]. Luminescence was recorded with the Fluostar Omega (BMG Labtech) plate reader.

#### Frozen sample preparation

Culture medium was removed and domes were washed with PBS at room temperature. Domes containing organoids were fixed with 4% PFA for 30 minutes at room temperature. After washing with PBS, domes were transferred to a 20% sucrose in PBS solution. Organoids were kept at 2–8°C until the domes fell to the bottom. Sucrose solution was removed and replaced with OCT Mounting media (VWR, 361603E). Frozen blocks were prepared using liquid nitrogen and stored at -20°C until sectioning. Organoid cryo-sections (5–10 μm-thick) were prepared using CryoStar NX70 cryostat (ThermoScientific).

#### Immunohistochemical and morphological analysis

All tissue samples were formalin-fixed, embedded in paraffin, sectioned using a microtome at a thickness of 5 μm and deparaffinised before further analysis. To evaluate the overall morphology and intestinal damage the slides were stained with haematoxylin and eosin (H&E), according to standard practice, then dehydrated and mounted. Murine organoid samples were also stained with H&E to conduct a histological evaluation. To detect apoptosis, the samples were stained with a TUNEL Assay Kit according to the manufacturer’s instructions. To detect cell proliferation, immunohistochemistry was performed using antibody and a horseradish peroxidase–DAB Detection IHC kit, following manufacturer´s guidelines detailed elsewhere [14]. In short, slides were deparaffinised, washed in PBS with Tween-20 and endogenous peroxidase activity was blocked using hydrogen peroxide. A DIVA-decloaking chamber was used for heat-induced antigen retrieval and non-specific background staining was blocked using the kit´s Protein Block solution [50]. Primary Ki67 antibody was added in a 1:1000 dilution of PBS-Tween, and incubated for one hour at 37°C. Slides were subsequently incubated for 10 min with biotinylated goat anti-rabbit antibodies and streptavidin peroxidase at room temperature. DAB was added to the jejunum tissue for 2 min and rinsed. Washing in between steps was done with PBS. Finally, slides were counterstained with methyl green, dehydrated, and mounted.

All images were acquired by a Zeiss Axio Vert microscope equipped with a Zeiss Axiocam 208 colour camera, Zeiss A-Plan 5x/0,25 Ph1 objective and Zeiss Zen Blue 4.3 software was used. To evaluate the overall morphology and intestinal damage, villus height and crypt depth were measured using Fiji ImageJ. After Ki67 staining, images were processed using in an ImageJ macro to automatically quantify the amount of DAB [14]. For TUNEL staining, the images were processed in ImageJ to quantify the number of stained cells per crypt by manually counting the number of stained cells in the crypt region per captured image and dividing by the number of crypts detected in the same image.

#### Confocal microscopy

The morphology and viability of organoids was visualized by confocal laser scanning microscopy (Carl Zeiss LSM 700 Laser Scanning Microscope, Jena, Germany). Organoid cultures were stained with the LIVE/DEAD™ Viability/Cytotoxicity Kit, for mammalian cells (ThermoFisher, L3224) and NucBlue™ Live ReadyProbes™ Reagent (Hoechst 33342) (ThermoFisher, R37605). Images were obtained using a Plan-Apochromat 10x/0.45 (Zeiss) objective. Organoids, were imaged with a pinhole setting of 2 Airy unit (14 μm sections) for every Z section over the total height where the organoids were distributed in the gels. To visualize 3-dimensional distribution, the Z stacks were post-process in maximal intensity projection with Fiji/Image J.

### Statistical analysis

The statistical data analysis was performed with an ANOVA analysis with Šidák’s multiple comparisons *post hoc* test, and comparisons with p<0.05 were considered significant. All comparisons were tested for normality of residuals and equality of group variance with the Shapiro-Wilk and Brown–Forsythe tests, respectively. If the group variance was not equal, the regular ANOVA analysis was replaced with a Brown-Forsythe ANOVA test with a Dunnet T3 *post hoc* test. If a non-normal distribution was indicated, the non-parametric Kruskal-Wallis test with Dunn’s multiple comparisons *post hoc* test was used.

For the murine organoids, the ROUT method, with a Q cut-off of 1%, was used to identify and exclude outliers in the data. One outlier was eliminated based on this threshold. The choice of a Q cut-off of 1% was based on previous experience with 3D cell culture and organoid models [51, 52], ensuring a balance between sensitivity and specificity [53]. Organoid viability data were plotted in GraphPad Prism and fitted with three-parameter inhibitor response curves using a least squares regression model [54]. An extra-sum of squares F-test and comparison of fits was used to calculate the statistical difference between IC_50_ values generated from best-fit lines, according to the software´s recommended approach [54].

All statistical tests and graphs were made in GraphPad Prism 9.0.0 (GraphPad Software, San Diego, CA, USA). Data is presented in the text as mean ± standard deviation. The sample size (n = 6) was selected based on previous experience of the effect and variability (body weight loss, villus height, and permeability) following single-dose chemotherapy treatment to rats.

## Results

**Fig 2:**
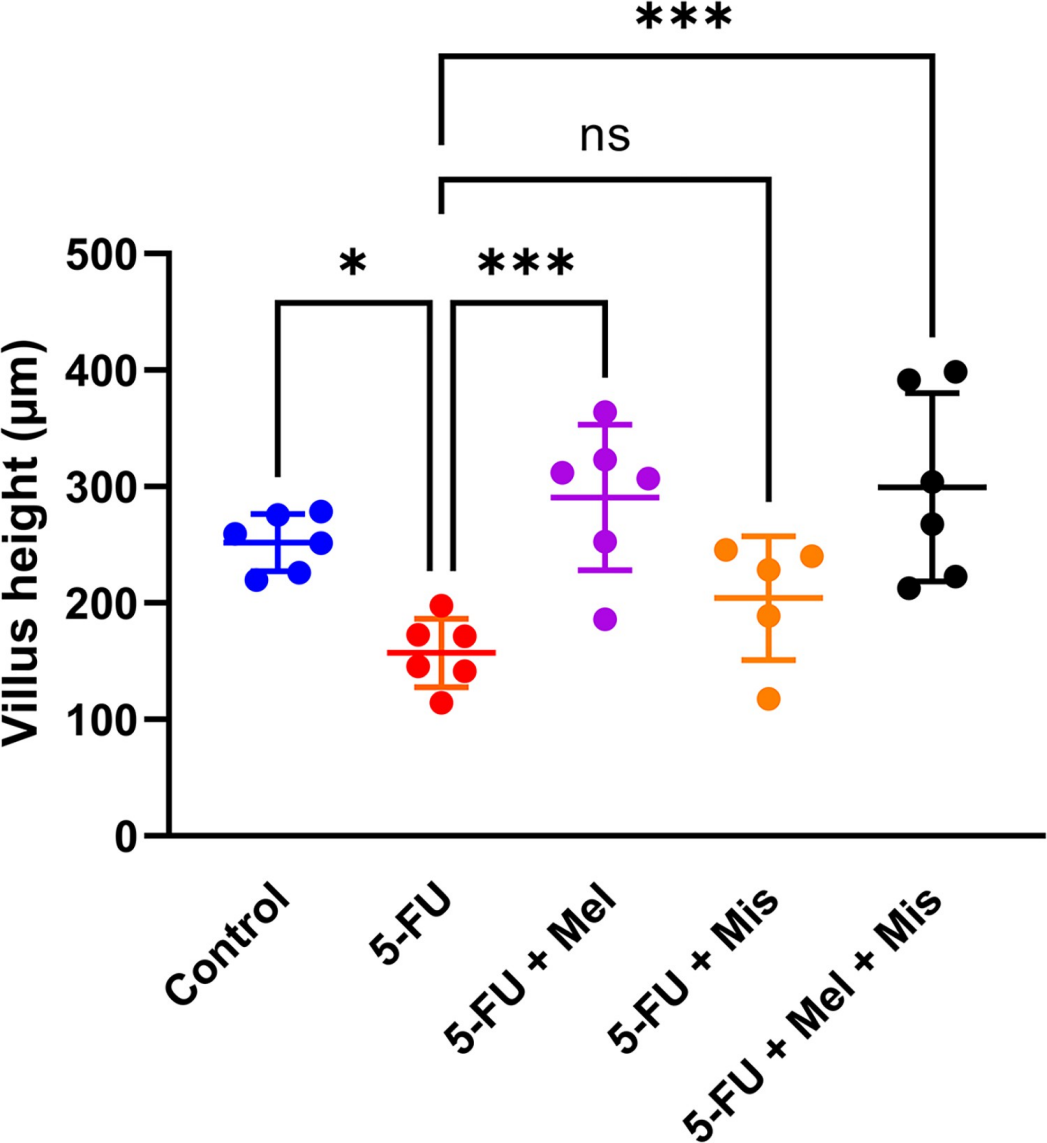
Preventive and treatment effects of melatonin (Mel, IP 10 mg/kg daily starting 24 h before 5-FU) and/or misoprostol (Mis, IP 1 mg/kg daily starting 24 h before 5-FU) on jejunal villus height following systemic exposure to 5-Fluorouracil (5-FU, IP 200 mg/kg). Each point represents the mean value from a single animal based on ten separate determinations, the short horizontal line signifies the group mean. The statistical data analysis was performed with an ordinary one-way ANOVA test with Dunnett’s multiple comparisons test, and significant comparisons were indicated by one (p < 0.05), three (p 0.001), or four (p < 0.0001) stars.

The jejunal villus height in rats was assessed following intraperitoneal (IP) daily treatments with misoprostol and/or melatonin, both with or without a single IP dose of 5-FU (200 mg/kg) to induce CIM. Samples were taken from all six experimental groups at 72 hours post-dosing (Fig 1). Histological assessment revealed that a single dose of 5-FU significantly reduced the villus height from 252 ± 25 μm (control) to 157 ± 29 μm (p < 0.05), as shown in Fig 2. In 5-FU induced rats the small intestinal villus height was after daily treatment with melatonin alone (291 ± 63 μm, p < 0.01) or in combination with misoprostol (300 ± 81 μm, p < 0.005). However, monotreatment with misoprostol in 5-FU induced rats had no significant effect on this villus height reduction (204 ± 53 μm).

### Programmed cell death *in vivo* in rats

**Fig 3:**
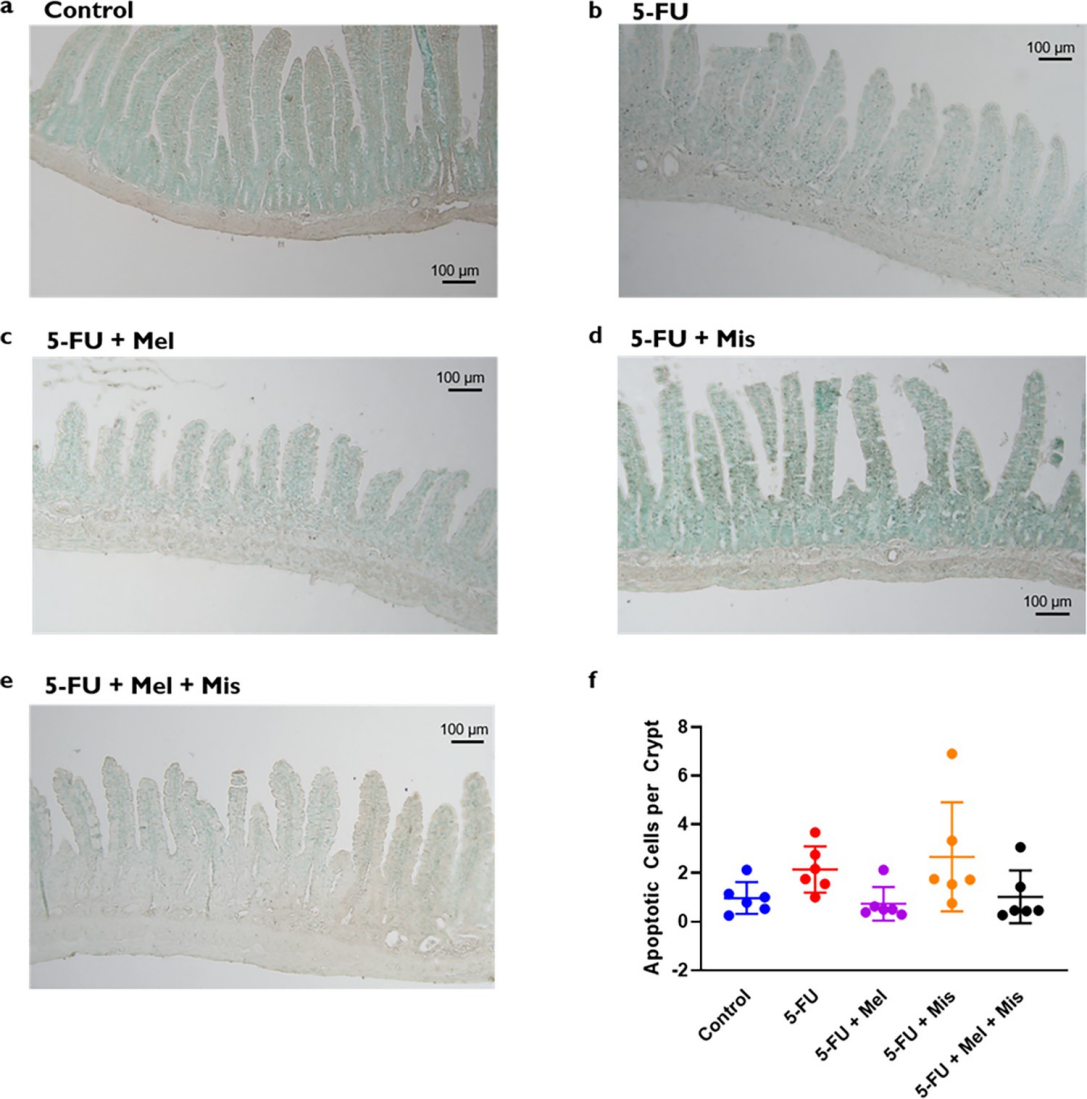
**a-f.** Apoptosis (number of apoptotic cells per crypt determined with TUNEL-positive staining) in jejunum samples 72 h after IP dosing of 5-Fluorouracil (5-FU, 200 mg/kg) and melatonin (Mel, IP 10 mg/kg daily) and/or misoprostol (Mis, IP 1 mg/kg daily). Representative tissue images for each study group are shown in Fig 3A-3E for saline (1 ml/kg Control), 5-FU only, and 5-FU with daily dosing (starting 24 h before 5-FU dosing) of melatonin, misoprostol, or melatonin and misoprostol. The results from the staining analysis are shown in Fig 3E, where each point represents the value from a single animal and the horizontal line signifies the group mean. The statistical data analysis was performed with a Kruskal-Wallis test with Dunn’s multiple comparison test. The different treatments were not significantly different from each other. Scale bars represent 100 μm.

Representative images of the TUNEL staining and the number of apoptotic cells per small intestinal crypt in rats at 72 hrs after single dose of 5-FU are displayed in Fig 3A–3E. In control animals, the average number of apoptotic cells per crypt was 1.0 ± 0.7 (Fig 3F). In CIM-induced animals administered 5-FU alone, the average number of apoptotic cells per crypt was slightly increased to 2.1 ± 0.9 compared to the control. In 5-FU CIM-induced animals melatonin reduced the average number of apoptotic cells per crypt to 0.7 ± 0.7 (p = 0.05). Neither the combination of melatonin and misoprostol or misoprostol along in 5-FU CIM-induced rats had no effect the average number of apoptotic cells per crypt as they were 1.0 ± 1.0 (p = 0.11) and 2.7 ± 2.2, respectively.

### Proliferation *in vivo* in rats

**Fig 4:**
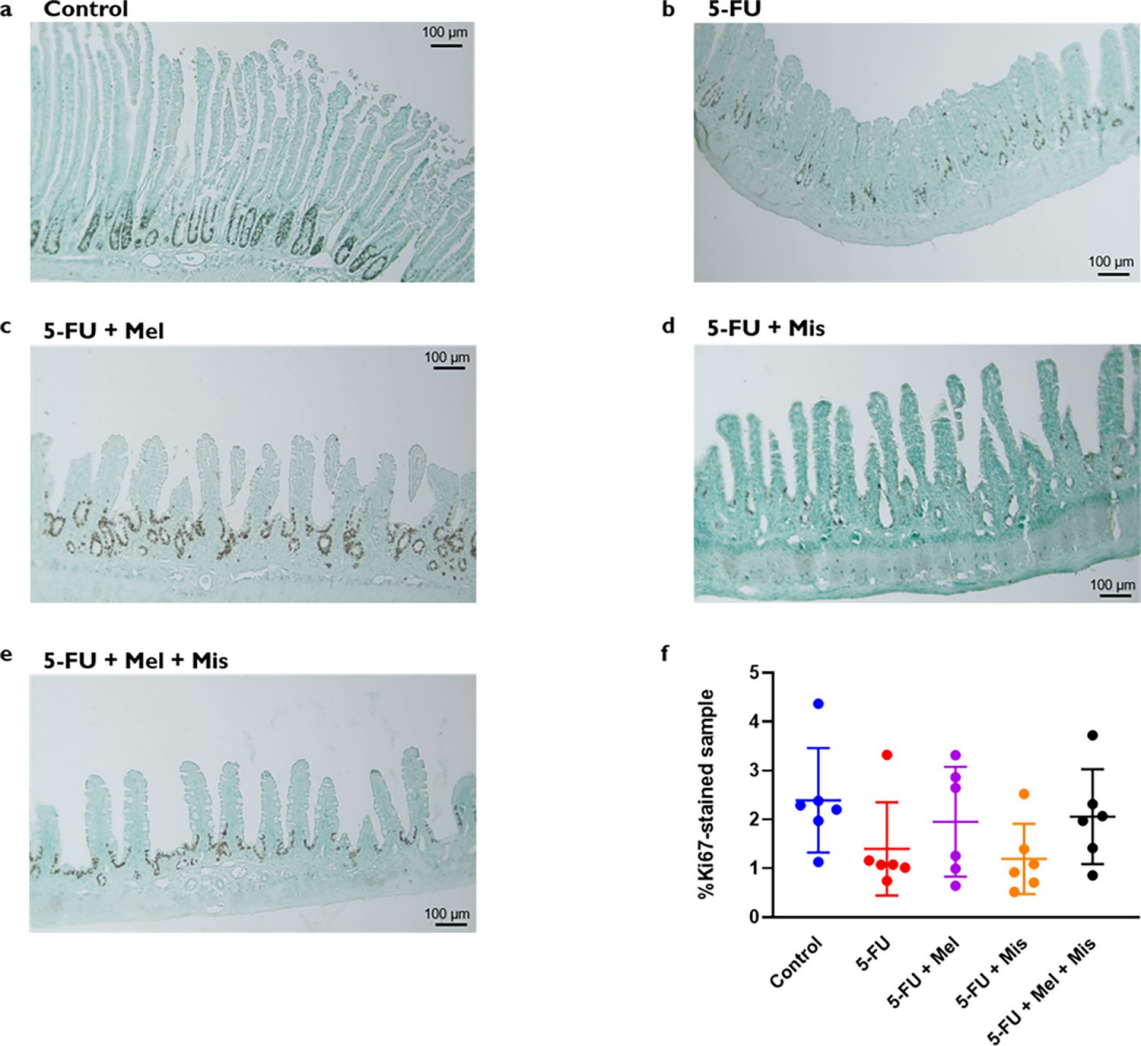
**a-f.** Proliferation (percentage of sample area stained for Ki67) in jejunum samples 72 h after IP dosing of 5-Fluorouracil (5-FU, 200 mg/kg) and melatonin (Mel, IP 10 mg/kg daily) and/or misoprostol (Mis, IP 1 mg/kg daily). Representative tissue images for each study group are shown in Fig 4A-4E for saline (1 ml/kg Control), 5-FU only, and 5-FU with daily dosing (starting 24 h before 5-FU dosing) of melatonin, misoprostol, or melatonin and misoprostol. The results from the staining analysis are shown in Fig 4E, where each point represents the value from a single animal and the horizontal line signifies the group mean. The statistical data analysis was performed with a Kruskal-Wallis test with Dunn’s multiple comparison test. The different treatments were not significantly different from each other. Scale bars represent 100 μm.

Representative images of the Ki67 staining and the percentages of Ki67-positive staining after the different IP daily treatments with misoprostol and melatonin, 72 hrs after a single IP dose of 5-FU, are shown in Fig 4A–4E. No significant differences were observed amongst the groups. The control (2.4 ± 1.1%) showed a slightly higher average in levels of proliferation when compared to animals subjected to 5-FU alone (1.4 ± 1.0%) or with misoprostol (1.2 ± 0.7%) (Fig 4F). Both groups given 5-FU and melatonin with (2.1 ± 1.0%) or without (1.9 ± 1.1%) misoprostol displayed averages close to the control (Fig 4F).

### Colonic faecal water content *in vivo* in rats

**Fig 5:**
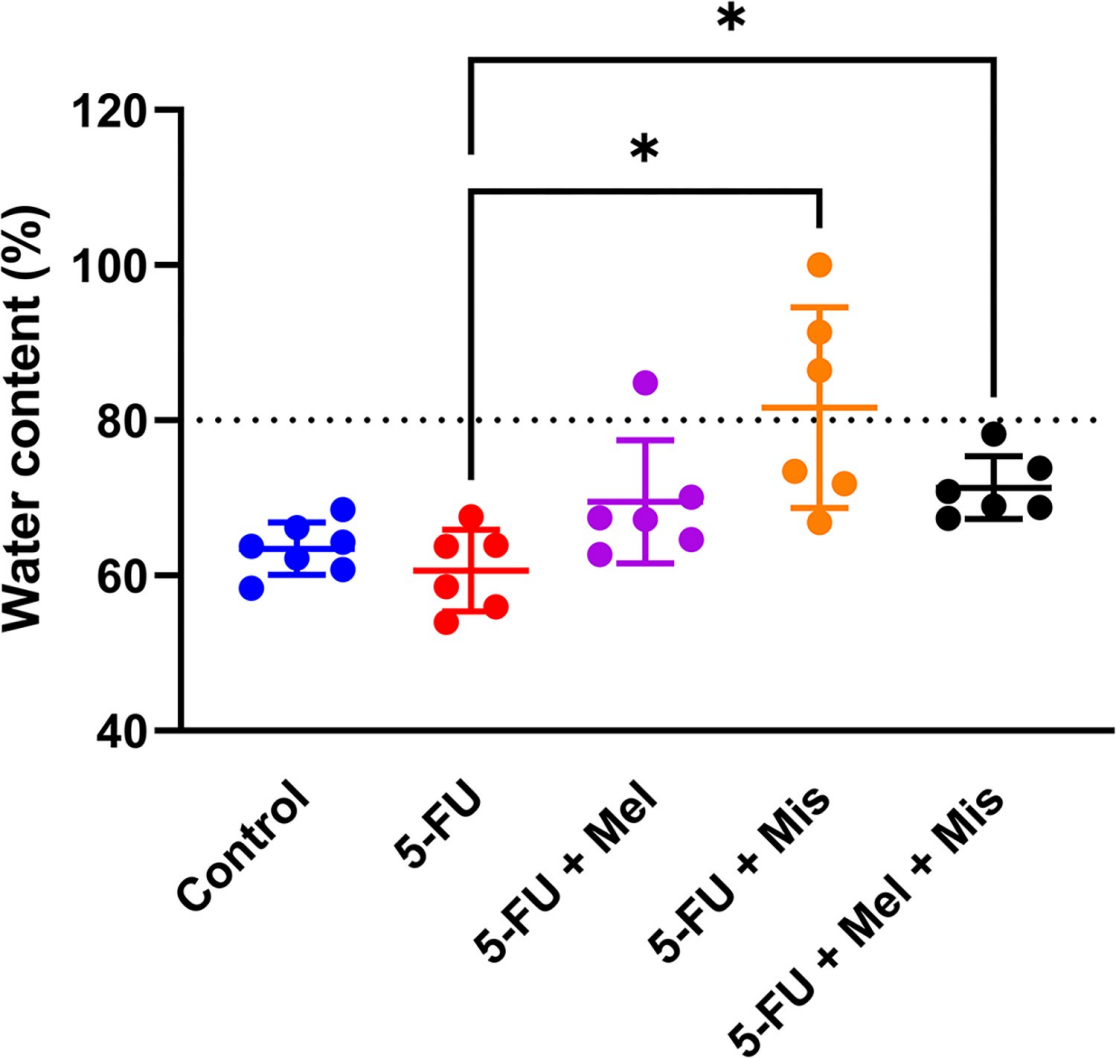
Colonic fecal water content (%) reflects diarrhea 72 h after dosing of 5-Fluorouracil (5-FU, 200 mg/kg) and melatonin (Mel, 10 mg/kg daily starting 24 h before 5-FU) and/or misoprostol (Mis, 1 mg/kg daily starting 24 h before 5-FU). Each point represents the percent water content from a single animal, the horizontal line signifies the group mean. The dotted line at 80% represents a threshold from which diarrhea can be assumed. The statistical analysis was performed with a Brown-Forsythe and Welch ANOVA test with Dunnett’s T3 multiple comparisons test. Comparisons with p < 0.05 were considered significant, indicated by one star.

The degree of diarrhea was determined by collecting fecal contents of the colon into plastic tubes. After drying, the water contents could be quantified, calculated and expressed as percent of water. The results at 72 hrs in all study groups are shown in Fig 5. The fecal water contents in untreated control animals were 63 ± 3%. A single dose of 5-FU (200 mg/kg) did not influence the fecal water content (61 ± 5%). In the same way, melatonin administration in animals with 5-FU-induced CIM did not significantly affect the fecal water content (69 ± 8%). Interestingly, all groups of animals subjected to 5-FU and misoprostol displayed a significant increase in fecal water content. When the combination of misoprostol and melatonin, or misoprostol alone was given, the water content was 71 ± 4% and 82 ± 13%, respectively. Both groups showed significantly higher fecal water content compared to the 5-FU subjected group (p < 0.05).

### Changes in body weight *in vivo* in rats

**Fig 6:**
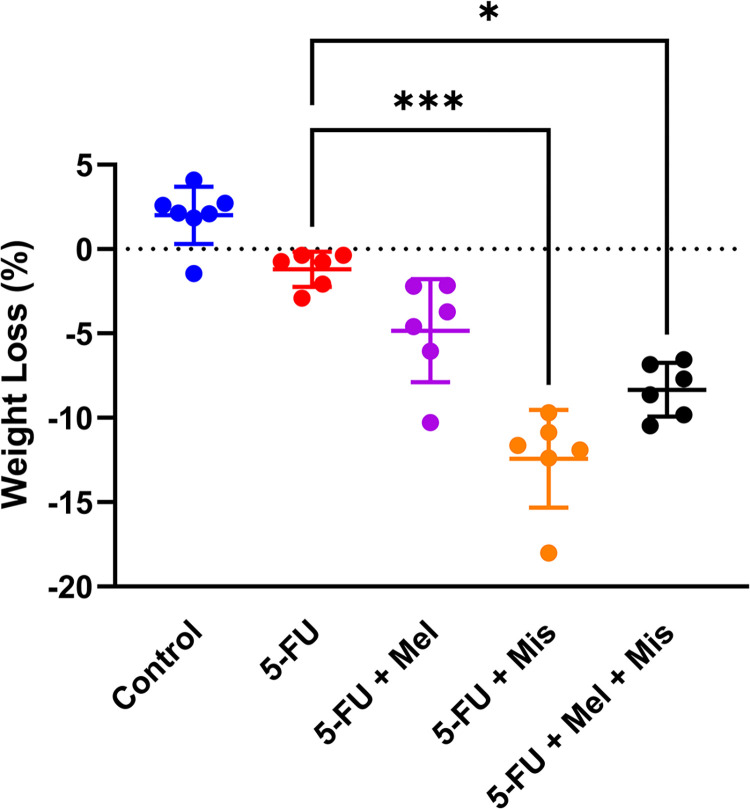
Body weight loss 72 h after dosing of 5-Fluorouracil (5-FU, 200 mg/kg) and melatonin (Mel, 10 mg/kg daily starting 24 h before 5-FU) and/or misoprostol (Mis, 1 mg/kg daily starting 24 h before 5-FU). Each point represents the percent water content from a single animal, the horizontal line signifies the group mean. The statistical data analysis was performed with a Kruskal-Wallis test with Dunn’s multiple comparison test. Comparisons with *p* < 0.05 were considered significant and significant comparisons were indicated with one (*p* < 0.05) or three (*p* < 0.001) stars.

Animals given 5-FU and misoprostol, either without or with melatonin, demonstrated a significant reduction in body weight by 12 ± 3% and 8 ± 2% respectively, compared to those treated with 5-FU alone (Fig 6). There were no statistically significant changes in body weight in any treatment group, as shown in Fig 6.

### MT_2_ melatonin receptor expression *in vivo* in rats

**Fig 7:**
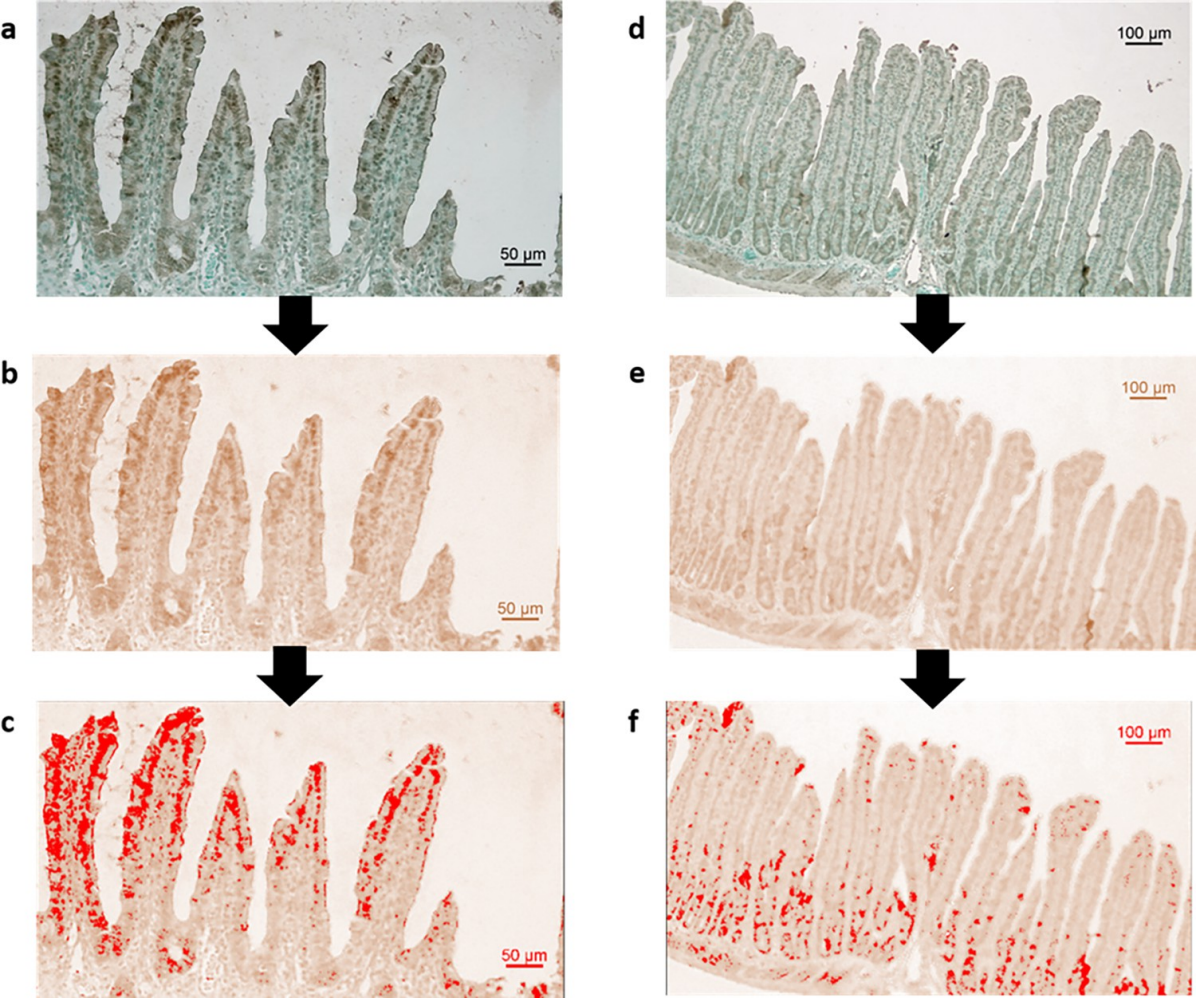
Expression of the MT_2_ melatonin receptor in rat jejunum. Animals administered 5-FU and melatonin (a-c) or 5-FU alone (d-f). Original microscopy pictures are shown in a and d. Color deconvolution was performed to split the different color channels and show only the DAB portion (b, e). Finally, a threshold was set to highlight the areas stained with a higher intensity (in red) from unspecific background staining and (c, f).

In rats administered 5-FU alone, the MT_2_ melatonin receptor was only prominently expressed in the crypt of the rat jejunum (Fig 7D–7F). In rats administered both 5-FU and melatonin, the MT_2_ melatonin receptor was shown to be prominently expressed in the villus part of the rat jejunum, which could suggest that melatonin has a protective effect for the villus cells (Fig 7A–7C).

### Murine *in vitro* intestinal organoids morphology

**Fig 8:**
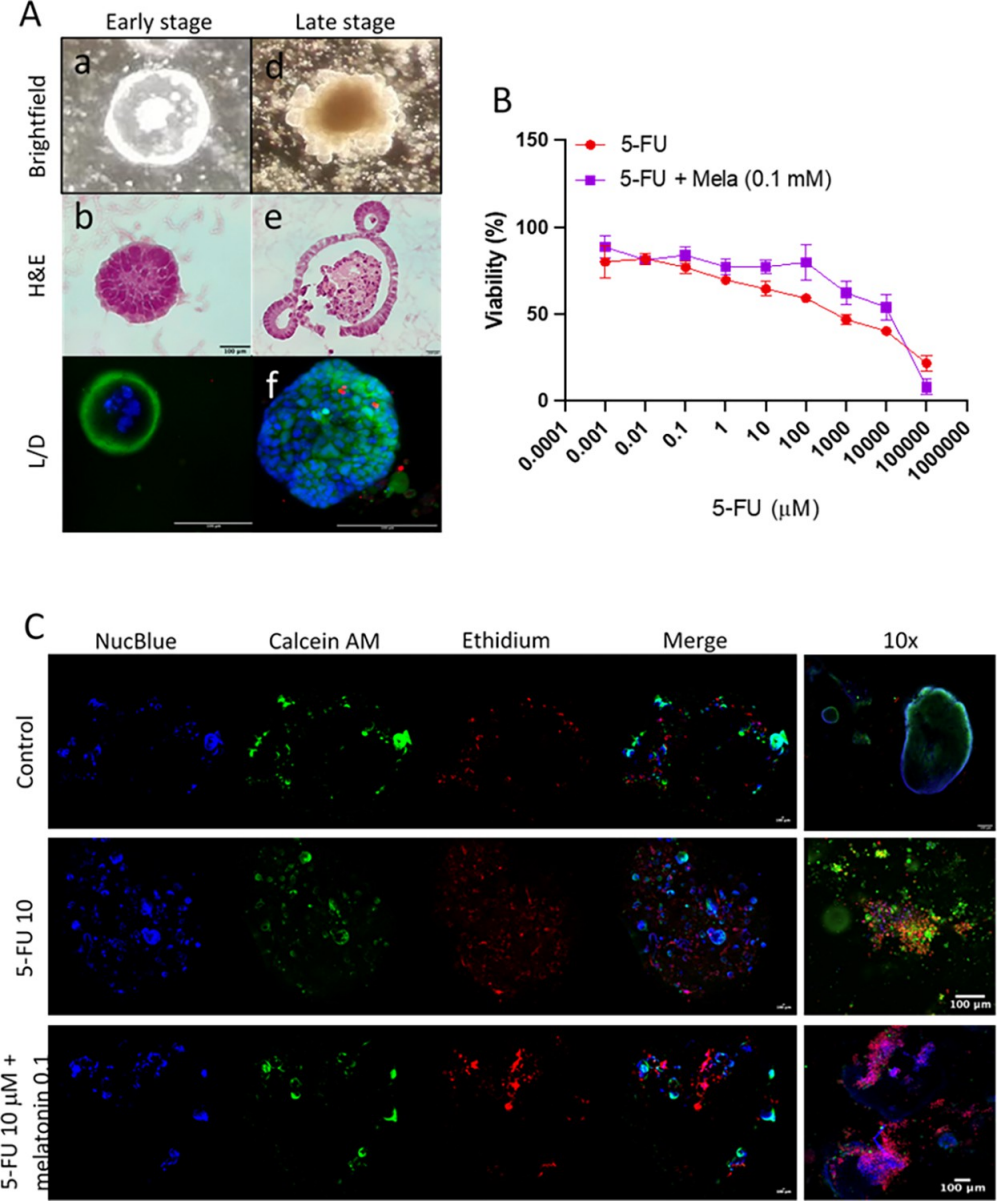
Murine intestinal organoids experiments. (A) In vitro growth of healthy murine intestinal organoids. Representative images showing early (a-c) and late (d-f) stages of development. Morphology and viability were assessed by light microscopy (a, d), haematoxylin and eosin staining (b, e) and fluorescent microscopy (c, f). (B) Cell viability curves of murine intestinal organoids treated with either 5-FU (0–100,000 μM) or 5-FU + melatonin (0–100,000 μM and 0.1 mM respectively). Data points represent the median value ± 95% CI (n = 2–4 organoids/treatment) from 3 independent experiments. (C) 3-D confocal immunofluorescence imaging of murine intestinal organoids embedded in BME. Representative images of organoids treated with either 5-FU (10 μM), 5-FU (10 μM) + melatonin (0.1 mM) or control. Blue = DAPI (nuclei), green = calcein (live cells), red = ethidium (dead cells). Scale bars = 100 μm.

Murine intestinal organoids derived from intestinal crypts were isolated and cultured in extracellular matrix (*in vitro*). We demonstrated the *in vitro* maturation from isolated crypts to cyst-like spheroid structures with a pseudo-lumen (Fig 8A, a-c). Eventually these morphological structures will be further developed into fully differentiated multi-lobular crypt-like organoids (Fig 8A, d-f).

### Effect of melatonin pre-treatment on 5-FU-induced cytotoxicity in *in vitro* murine intestinal organoids

Murine intestinal organoids were exposed to 5-FU in the concentration range 1 x 10^−4^–1 x 10^6^ μM alone or in combination with 0.1 mM melatonin. The effect of 5-FU on cell viability was concentration-dependent, resulting in a calculated IC_50_-value for cytotoxicity of 1190 ± 67 μM (Fig 8B). Interestingly, when 5-FU was combined with 0.1 mM melatonin, a significant mitigation of 5-FU-induced cytotoxicity was observed, elevating the IC_50_ value more than 10-fold (Fig 8B). This marked reduced cytotoxicity for 5-FU suggests that melatonin, when administered alongside 5-FU, may exert a protective effect against 5-FU-associated cytotoxicity on intestinal epithelial cells.

### Mucosal morphology *in vitro* in murine intestinal organoids

To assess the morphological changes in intestinal organoids following treatment, live/dead staining and confocal live imaging were conducted (Fig 8C). Immunofluorescence images revealed distinct morphological differences among the treatment groups. While the majority of organoids in the control group exhibited normal morphology, there was an elevated presence of cell death staining in 5-FU-treated organoids. Notably, the addition of melatonin reduced this staining (Fig 8C), suggesting a mitigating effect on 5-FU-induced cytotoxicity which is in accordance with its ability to increase the IC_50_ value.

## Discussion

Cancer treatment involves a range of therapies designed to eliminate or control abnormal cell growth. In chemotherapy, drugs are used to kill cancer cells and/or stop their growth. However, chemotherapeutic agents also have cytotoxic effects on healthy rapidly dividing cells [24]. Chemotherapy-induced mucositis (CIM) is a common side effect of cancer treatment. In severe cases the intestinal mucositis and the disrupted normal function of the intestinal epithelium results in difficulty in maintaining proper nutrition and hydration, leading to delays in cancer treatment and decreased quality of life for the patient. Breakdown of the epithelial barrier may also lead to sepsis and multi-organ failure [55].

This study investigated the effects of melatonin and misoprostol on mitigating the side effects of 5-fluorouracil (5-FU), specifically focusing on CIM and small intestinal mucosal morphology and atrophy. We observed a 38% reduction in villus height after a single dose of 5-FU (200 mg/kg), values consistent with previous reports of cytostatic-induced mucosal damage [15, 17]. Melatonin administration (10 mg/kg, once daily for four days) countered this atrophy. Moreover, melatonin’s protective role extended beyond structural preservation, (p = 0.05), reduced the number of apoptotic cells in the intestinal epithelium, suggesting its potential in modulating the cytotoxic effects of 5-FU. This was complemented by *in vitro* findings where melatonin notably increased the IC50 by a ten-fold in murine intestinal organoids exposed to 5-FU, indicating a protective effect against 5-FU-induced cytotoxicity.

Our findings suggest that melatonin’s roles as a reactive oxygen species (ROS) scavenger and an anti-inflammatory agent, could be of significance in mitigating CIM. Contrary to misoprostol, which did not exhibit a combined protective effect against villus atrophy, melatonin alone showed promise in protecting against CIM. A notable aspect of our study involved examining apoptosis in the small intestinal epithelium, a critical factor in villus atrophy and CIM development [16, 56, 57]. Post-chemotherapy, both human and animal models exhibit a surge in apoptotic cells, often peaking as early as 24 hours after treatment [16, 56, 57]. Our observations revealed a slight increase in apoptotic cells in the crypt region of the jejunal mucosa, 72 hours following 5-FU administration, but these levels were not statistically significant. This suggests that the peak impact of 5-FU on apoptosis could have occurred at an earlier point. Notably, all animals receiving 5-FU and melatonin exhibited fewer apoptotic cells compared to those administered 5-FU alone and had the same number of apoptotic cells as control animals, suggesting that melatonin inhibits 5-FU-induced increases in apoptosis. Intriguingly, in our murine intestinal organoids, melatonin notably reduced 5-FU-induced cell death, highlighting its potential to modulate apoptosis and enhance cellular survival, at least in the acute phase after injury.

Additionally, it has been shown that the rate of proliferation of small intestinal epithelial cells is another key factor contributing to CIM [7, 24]. A profound decline in area stained for the proliferation marker Ki67 has been reported 24 h after doxorubicin or irinotecan dosing, values that return to baseline 72 h after administration [16, 58]. The Ki67 stained area was not significantly different between controls and any other study group 72 h after 5-FU administration. This implies that the observed protective effects of melatonin might be more attributable to reduced apoptosis at earlier stages rather than an increase in proliferation [58]. Future investigations should, therefore, focus on earlier time points to comprehensively understand how melatonin mitigates apoptosis induced by 5-FU, providing a more complete picture of its protective mechanism against CIM.

To evaluate the possibility for melatonin to act in a protective manner not only through its role as a ROS scavenger [36], but also through receptor-mediated processes, we investigated the expression of the MT_2_ melatonin receptor in the intestinal mucosa. While we were able to show the presence of the receptor both in 5-FU groups treated with or without melatonin, the expression pattern appears to differ slightly between the two. While it was mostly expressed in the crypts in animals administered 5-FU alone, the addition of melatonin resulted in a shift of expression to the villus tips. The difference in expression may suggest that administration of melatonin protect the tissue and the enterocyte and villus mature and their normal physiological functions are preserved. Furthermore, the change, or translocation, of the MT_2_ melatonin receptor to the villus tips could be associated with induced net fluid and electrolyte transport, highlighting a potential mechanism for melatonin’s protective effects on the intestinal mucosa [59].

Misoprostol, binding to G-protein-coupled prostaglandin E receptors 1–4, have been reported to protect against intestinal mucosal damage and is clinically used for preventing nonsteroidal anti-inflammatory drug-induced ulcers [39, 60, 61]. It exerts cytoprotective effects by reducing pro-inflammatory cytokines like IL-1, IL-6, IL-8, and TNF, and inhibiting acid secretion while activating cellular survival pathways [38, 62]. Despite these properties, our study found no synergistic or any additive effect of misoprostol in combination with melatonin in reducing 5-FU-induced villus atrophy. Notably, misoprostol (1 mg/kg) led to increased colonic water content and significant body weight loss in animals, indicating severe diarrhea–a well-known side effect [63]. In addition, only animals treated with misoprostol significantly reduced body weight in this study further supporting a severe diarrhea in these animals. Overall, this data suggests that misoprostol would not be a suitable protective agent against CIM in cancer patients.

In conclusion, our research suggests melatonin as a potential agent for preventing CIM as well as intestinal tissue repairmen, advocating for its inclusion in cancer treatment regimens. Melatonin was effective in mitigating 5-FU-induced small intestinal villus atrophy in rats, as well as increasing cell viability in murine intestinal organoids exposed to 5-FU. This warrants further exploration of the mechanisms of the protective effect of melatonin at earlier (24 h for apoptosis and proliferation) and later (96 h for proliferation) time points. While misoprostol did not appear to be a viable future candidate, the promising results with melatonin in this study encourage further investigations in animals with cancer during longer treatment periods and different doses to form the basis for a translation to human cancer patients. As melatonin is suggested to display anti-cancer effects of its own, it is a promising candidate for synergistic cancer therapy, especially in a well-designed oral controlled release formulation that will expose and protect along the intestine.

## Supporting information

S1 File Raw data for villus height, apoptotic cells, %Ki67 stained sample, Water contents (%), and Weight loss (%). (XLSX)

S2 File Raw data for organoids viability. (XLSX)
